# Negative Impact of COVID-19 Upon Primary Brain Tumor Care

**DOI:** 10.7759/cureus.17800

**Published:** 2021-09-07

**Authors:** Gurpreet Sarwan, Taufif Mubarak, Persis Puello, Michael Brisman, Jai Grewal

**Affiliations:** 1 Physical Medicine and Rehabilitation, Nassau University Medical Center, East Meadow, USA; 2 Anesthesiology, Mount Sinai Hospital, Oceanside, USA; 3 Family Medicine, Mount Sinai Hospital, Oceanside, USA; 4 Neurosurgery, Mount Sinai Hospital, Oceanside, USA; 5 Neuro-oncology, Mount Sinai Hospital, Oceanside, USA

**Keywords:** paliative care, covid 19, sars-cov-2, hepatic tumors, recurrent meningioma

## Abstract

Meningiomas are the most common primary central nervous system tumors, as they can account for up to one-third of all primary brain tumors. Most meningiomas are benign, although up to one-fourth of such tumors are classified as atypical or malignant. Atypical and malignant meningiomas are associated with an increased risk of local recurrence and decreased overall survival. Our patient is a 57-year-old male with a history of recurrent malignant meningioma, with metastasis to the liver. He underwent multiple surgical interventions, radiation treatments, and systemic therapies for a malignant meningioma, ultimately requiring transfer to hospice care. Not only did a positive novel coronavirus (COVID-19) infection delay his ability to receive radiation therapy, the infection in itself may have had an impact on the course of care for this patient. Treatment targeting the patient’s COVID-19 infection may have suppressed the immune system, and as a result, caused the progression of metastatic disease. Palliative care was needed in the setting of losing all functional goals for quality of life due to malignant neoplasm.

## Introduction

During the COVID-19 pandemic, assessing the safety and utility of continuing cancer treatments continues to be challenging for providers and cancer patients. This case report reviews a 57-year-old male with a recurrent malignant meningioma, with metastasis to the liver. The patient was beginning a new round of radiation therapy for his meningioma when it was noted he had a fever and needed further evaluation. It was found that his disease course was further complicated by novel coronavirus disease 2019 (COVID-19) infection. A recent study conducted by the University College of London has shown that severe acute respiratory syndrome coronavirus 2 (SARS-CoV-2) infection can cause an increase in delirium and brain inflammation [1]. This may have further compounded the clinical presentation in our patient. This case report reviews the novelty of treating a COVID-19 positive patient with metastatic cancer, treatment safety for cancer patients during the COVID-19 pandemic, and the possible effect the virus has on the prognosis of cancer patients actively receiving treatment. 

A meningioma is typically a benign neoplastic growth originating from the leptomeninges. These tumors are the most common central nervous system (CNS) neoplasms, accounting for 36.4% of all primary brain and CNS tumors [2]. The World Health Organization (WHO) has developed staging criteria to further classify meningiomas into grade I, grade II, and grade III. Grade I meningiomas are considered benign, grade II meningiomas are labeled atypical, and grade III meningiomas are malignant [3]. Malignant meningiomas with distant metastases are rare. According to population-based studies, it is estimated that the incidence of grade III meningiomas is approximately one to three percent of all meningiomas [2]. 

Our patient is a 57-year-old male with a history of recurrent malignant meningioma, with metastasis to the liver. He underwent multiple surgical interventions, radiation treatments, and systemic therapies for a malignant meningioma, ultimately requiring transfer to hospice care. He had a superimposed infection by COVID-19, which was severe enough to require hospitalization. His hospitalization for COVID-19 impacted the course of medical care and possibly symptomatic progression of his disease. COVID-19 infection had an impact in delaying the patient’s radiation therapy. Treatment targeting the patient’s COVID-19 infection may have suppressed the immune system, and as a result, cause the progression of metastatic disease. Palliative care was needed in the setting of losing all functional goals for quality of life due to malignant neoplasm.

## Case presentation

This case involves a 57-year-old male who presented to his primary care provider (PCP) complaining of proximal right upper extremity (RUE) and right lower extremity weakness (RLE). Imaging confirmed at that time a WHO grade 1 left parietal meningioma, which was surgically resected. Four years later, the patient was noted to have tumor recurrence adjacent to his prior resection site and underwent a second left parietal craniotomy with re-resection of recurrent left frontoparietal mass (Table 1).

**Table 1:** Oncological intervention events IMRT - intensity-modulated radiation therapy, SRS - stereotactic radiosurgery

Month/year	Surgical intervention	Radiation therapy/radiosurgery	Systemic therapy
08/2009 surgical intervention #1	Left parietal craniotomy for meningioma resection	N/A	N/A
06/2013 surgical intervention #2	Left frontoparietal craniotomy for meningioma resection	N/A	N/A
01/2014 radiation therapy #1	N/A	IMRT-based limited field radiotherapy, 56Gy in 28 fractions	N/A
05/2015 radiation therapy #2	N/A	Gamma Knife SRS, 16Gy single fraction	N/A
07/2017 radiation therapy #3	N/A	IMRT-based SRS, Novalis TX, 550 cGy x 3 fractions	N/A
12/2017-05/2019 systemic therapy # 1	N/A	N/A	Sutent 37.5 mg 28 days on 14 days off
05/2019 systemic therapy # 2	N/A	N/A	Sandostatin LAR 30mg IM every 28 days
09/2019 surgical intervention #3	Partial craniotomy with cytoreduction	N/A	N/A

The patient underwent and completed radiation therapy. Upon completion, the patient reported improved right-sided strength, with minor residual weakness to his right hand. A repeat MRI showed a smaller left parietal mass, but a section of the right falx became slightly larger.

About eight months later, the patient underwent single-fraction stereotactic radiosurgery (SRS) procedure with Gamma Knife, targeting the falx and parietal region (Table 2).

**Table 2:** COVID-19 treatment IVPB - intravenous piggyback, NS - normal saline, CPAP - continuous positive airway pressure therapy, BiPap - bi-level positive airway pressure, IVP - intravenous pyelogram

Date	Treatment
First	Admission
5/4	Vancomycin IVPB 1500mg/500mL once
5/4-5/6	Vancomycin IVPB 1000mg/250mL every 12 hours, piperacillin + tazobactam 3.375 g in 500 mL NS every 8 hours
5/5-5/9	Enoxaparin 80 mg every 12 hours, ascorbic acid 1g daily, zinc sulfate 220mg daily, acetaminophen 650mg every 6 hours as needed
Second	Admission
5/11	Hydroxychloroquine 400mg once; CPAP day 1.
5/12	1 unit convalescent plasma; BiPap day 2, day 3 nights only through day 6
5/11-5/18	Piperacillin + tazobactam 3.375 g in 500 mL NS every 8 hours
5/11-5/27	Ascorbic acid 1g daily, zinc sulfate 220mg daily, enoxaparin 1mg/kg every 12 hours
5/14-5/15	Methylprednisolone 80mg IVP every 12 hours
5/15-5/18	Vancomycin 1g every 12 hours

**Figure 1:**
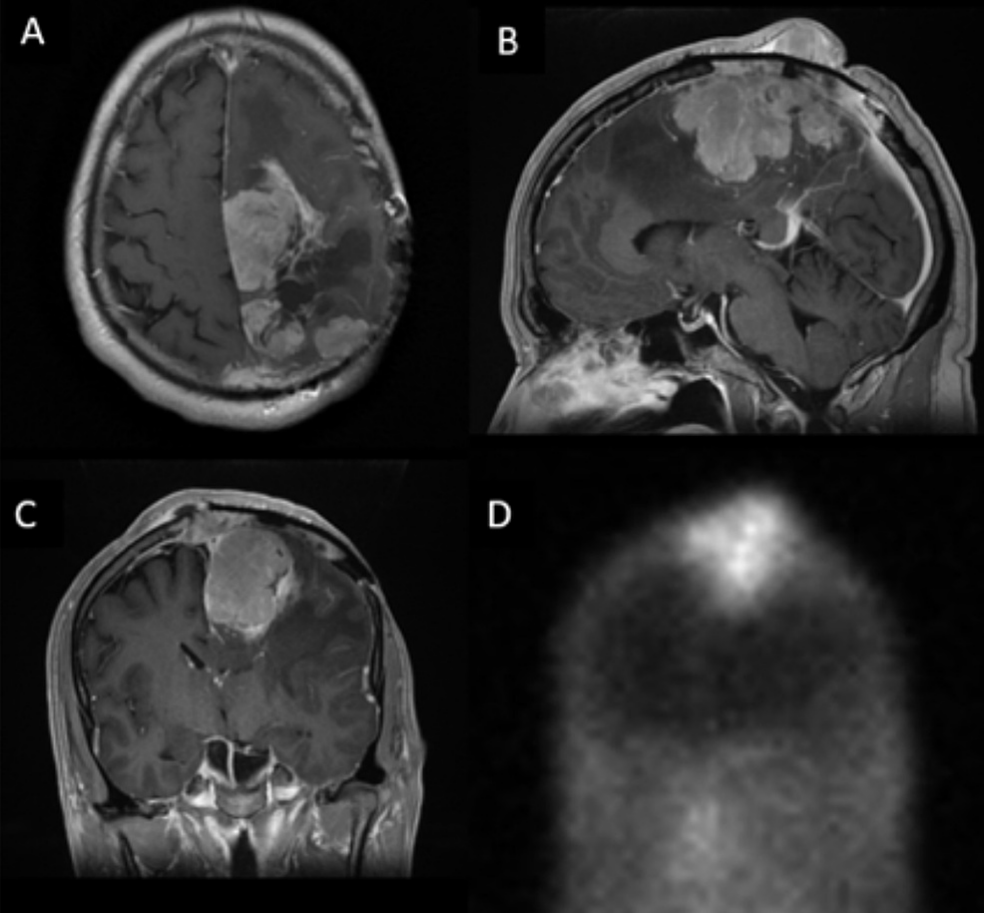
Magnetic resonance imaging T1-weighted contrast-enhanced MRI images in axial, sagittal, and coronal planes demonstrate a large left parietal meningioma with significant mass effect and invasion of the superior sagittal sinus and skull (A, B, C).  Nuclear medicine study demonstrates radiolabeled octreotide uptake within tumor on the coronal view (D).

Approximately one year later, the patient started to develop worsened RLE weakness. MRI showed a tumor impinging upon the motor strip just adjacent to the falx. At this point, fractionated SRS with Novalis-TX was administered. Several months later, the patient continued to have RLE weakness, impairing his gait. Repeat MRI at this time showed progression of the disease progressing through the skull and into the scalp. He was then started on Sutent chemotherapy based on a phase two trial [4]. He experienced radiographic and clinical responses that lasted approximately 18 months, by which time his RLE again became weaker in the setting of radiographic progression. Somatostatin receptor scintigraphy was performed, showing marked uptake into the tumor (Figure 1), and intramuscular octreotide LAR 30mg was administered every 28 days with four months of stability. At progression, he underwent a third surgical resection confirming WHO grade 1 meningioma with a low mitotic index (0-5%).

Approximately eight months following his final surgery, the patient reported abdominal pain, and imaging showed a hepatic mass. Imaging of the brain showed a new large right frontal mass. Biopsy of the liver lesion confirmed metastatic (anaplastic) meningioma WHO grade III. During his hospitalization, the patient was found to have a fever. The patient was tested for COVID-19 and was found to be positive. As a result, the patient was unable to begin radiation therapy and was admitted to the inpatient medicine floor for a prolonged period for management of COVID-19. During this time, the patient’s neurological status deteriorated at a rapid rate, ultimately involving palliative care and transfer to a hospice facility.

## Discussion

Meningiomas are the most common primary central nervous system (CNS) tumors and account for up to one-third of all primary brain tumors. Most meningiomas are benign, otherwise classified as WHO grade I. However, there is a certain subset that is atypical (WHO grade II) or malignant (WHO grade III), which represent a higher-grade tumor with a faster rate of progression and a higher rate of recurrence [3]. Accordingly, the quality of life can be impacted by atypical and malignant meningiomas, which are associated with a decreased overall survival. This case involves a 57-year-old male who initially presented with a WHO grade I benign meningioma. Despite multiple surgical, radiation, and systemic treatments, this patient’s meningioma progressed into a malignant form that metastasized into the liver. At this point, the plan of care was to begin radiation therapy. However, the patient was diagnosed with COVID-19, which required an extensive treatment course to recover from, ultimately leading to delayed treatment for this patient’s advancing meningioma.

COVID-19 has been a difficult virus to battle and has created a global pandemic leading to many lives lost. The difficulties lie in the limited research on treatment for this deadly disease. There have been many issues surrounding COVID-19, and an important topic of discussion is the treatment of cancer patients with superimposed COVID-19 infection, as our patient endured. For cancer patients diagnosed with COVID-19, there are no definitive guidelines on the treatment protocol, including when to restart therapy [5]. According to an updated American Society of Clinical Oncology guidelines, they are in favor of waiting until at least one SARS-CoV-2 test is negative before reinitiating anticancer therapy [6]. According to the Centers for Disease Control (CDC), an alternative non-test-based strategy is to resume therapy no sooner than 20 days after symptom onset (or, for asymptomatic patients, 20 days after the initial positive SARS-CoV-2 test) [7]. This is the basis for treatment for patients with severe or critical illness or who are immunocompromised. These guidelines continue to adjust as we learn more about the spread of infection, as well as the evolution of the virus itself. At this time, there is a lack of data exploring the benefit of any COVID-19 treatment in patients with cancer and SARS-CoV-2 infection.

Our patient was hospitalized twice due to COVID-19 pneumonia in May of 2020. This patient endured an extensive treatment course, outlined in Table 1, including antibiotic therapy with vancomycin, piperacillin/tazobactam, and hydroxychloroquine. This patient also received convalescent plasma, which is thought to use donor antibodies to possibly suppress the virus and modify inflammatory response [8]. The patient was also trialed on tocilizumab, an interleukin-6 inhibitor thought to treat a cytokine storm induced by the virus [9]. Current research explores other treatment options including ivermectin, which was not a medication commonly being trialed at the time of this patient's hospitalization [10]. There is limited research on how these therapies may impact the course of cancer progression. There is even a potential for causing a negative impact on cancer progression. In addition, during the course of this patient’s hospitalization, the patient had to delay radiation therapy. This, along with the COVID-19 superimposed infection, may have led to the decline in our patient’s prognosis. Our patient developed quadriparesis and right lateral gaze palsy, ultimately leading to loss of all functional goals for a decent quality of life. The decision was made to not pursue further treatment for the meningioma. After involving palliative care services, transfer to hospice was initiated. Since the start of the COVID-19 pandemic, there have been changes to the management. The management depends on the severity of the disease, which is ultimately decided upon symptoms, laboratory markers (including inflammatory markers), hypoxia and oxygen supplementation. In severe disease, which requires hospitalization, the management may include other medications such as dexamethasone [5]. Research continues to adapt and management continues to evolve; the current treatment strategies and protocol may have brought about a different outcome in our patient.

## Conclusions

This case represents an important problem that is being seen during the COVID-19 pandemic: if a cancer patient with a late-stage disease or with significant comorbid health conditions acquires severe COVID-19 and requires mechanical ventilation, the prognosis is likely to be dismal. Accordingly, it is important to have this discussion with the patient and their family and have palliative care specialists involved in the case to help facilitate the course of action. Depending on state regulations, patients should be offered the option of completing a Physician Order for Life-Sustaining Treatment (POLST) form and/or other types of out-of-hospital do not resuscitate (DNR) order, especially if they would not want to receive cardiopulmonary resuscitation (CPR) or mechanical ventilation. Consultation with a palliative care specialist is beneficial for having these difficult conversations with patients and their families. Potential questions to address: does COVID-19 accelerate the need for hospice? How can we navigate palliative care issues for patients with advanced-stage meningioma vs. COVID-19? Another important consideration to take into account would be whether or not the management of COVID-19 could have changed the outcome in our patient, seeing how COVID-19 treatment strategies continue to shift as research evolves.
